# Metabolomics of Chlorophylls and Carotenoids: Analytical Methods and Metabolome-Based Studies

**DOI:** 10.3390/antiox10101622

**Published:** 2021-10-15

**Authors:** María Roca, Antonio Pérez-Gálvez

**Affiliations:** Food Phytochemistry Department, Instituto de la Grasa (CSIC), Building 46, 41013 Sevilla, Spain; mroca@ig.csic.es

**Keywords:** antioxidants, carotenoids, chlorophylls, extraction methods, novel analytical technologies, metabolomics, mass spectrometry, metabolism, pathways, pigments

## Abstract

Chlorophylls and carotenoids are two families of antioxidants present in daily ingested foods, whose recognition as added-value ingredients runs in parallel with the increasing number of demonstrated functional properties. Both groups include a complex and vast number of compounds, and extraction and analysis methods evolved recently to a modern protocol. New methodologies are more potent, precise, and accurate, but their application requires a better understanding of the technical and biological context. Therefore, the present review compiles the basic knowledge and recent advances of the metabolomics of chlorophylls and carotenoids, including the interrelation with the primary metabolism. The study includes material preparation and extraction protocols, the instrumental techniques for the acquisition of spectroscopic and spectrometric properties, the workflows and software tools for data pre-processing and analysis, and the application of mass spectrometry to pigment metabolomics. In addition, the review encompasses a critical description of studies where metabolomics analyses of chlorophylls and carotenoids were developed as an approach to analyzing the effects of biotic and abiotic stressors on living organisms.

## 1. Introduction

Metabolomics is an essential approach that allows for the acquisition of knowledge regarding the actual composition of complex mixtures of extracts from tissues of plant or animal origin. The development of metabolomics is only feasible with a holistic methodology, applying a multifaceted and interdependent sequence of experiments, techniques, and computational tools [1]. Accordingly, the successful application of metabolomics depends on the successful selection or development of extraction protocols; the arrangement of the suitable analytical platform for analyses; the implementation of software for data gathering, handling, and analysis of results, where an expert-curated learning attitude is fundamental; and, finally, the application of statistics to extract the information within a biological context [2]. Nevertheless, the importance of metabolomics lies in the information regarding the physiology of an organism, tissue, cell, etc. Indeed, metabolomics is a source that reflects a biochemical state or activity.

**Figure 1:**
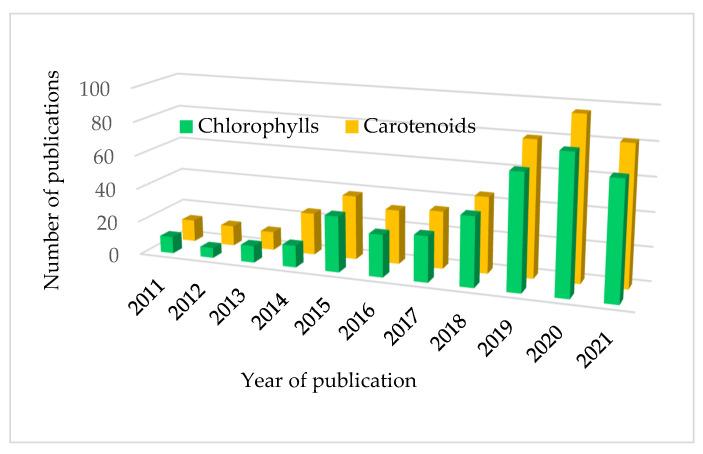
Number of publications since 2011 in the Web of Science (ISI Web of Knowledge) databases, introducing “metabolomic*” and “chlorophyll*” (green series) and “metabolomic*” and “carotenoid*” (orange series) as topics. The year 2021 does not cover the whole year and takes into account publications from January to September only.

This review is focused on the metabolomics of chlorophylls and carotenoids, which was named “pigmentomic”, as a tool for exploring their antioxidant features within the secondary plant metabolism. The antioxidant properties of both families of pigments have been deeply investigated and recently reviewed [3]. To gain an idea of the present impact of this topic, we performed a reference search in the Web of Science (ISI Web of Knowledge) databases, introducing “metabolomic*” and “chlorophyll*” as topics, and a total of 380 results were obtained (Figure 1). Moreover, when the topics “metabolomic*” and “carotenoid*” are selected, 499 results arise. However, the interesting point of both surveys is their time evolution, as half of the manuscripts were published in 2019 or thereafter, a clear signal of the exponential growth rate of metabolomics studies focusing on chlorophylls and carotenoids. This review includes metabolomics and metabonomics studies, as the difference between both terms is author dependent, and each term was defined as a subset of the other. It can be assumed that in metabolomics (stated by Fiehn and collaborators in 2001) [4], studies are necessary to identify and quantify all endogenous metabolites, while the metabonomics assessment (created by Nicholson et al. in 1999) [5] aims to identify a metabolite fingerprint. In a broad sense, a metabolomics strategy utilizes a mixture of separation techniques, such as HPLC or GC-MS, while in metabonomics studies, the use of NMR spectroscopy is more frequent.

We summarize the current understanding of how metabolomics describes fluctuations in chlorophylls and carotenoids, which perform essential functions and actions in photosynthetic organisms and animals that incorporate them through diet. Their involvement in plant biochemistry as key network components suggests that chlorophylls and carotenoids are key compounds involved in significant metabolic pathways. The review starts with a general description of the application of techniques for sample preparation and the acquisition of extracts suitable for analysis; a picture of the analytical platform and technologies applied for the identification and quantification of the pigment profile; and the workflow for data analysis, including software tools and the application of metabolite databases and statistics. Next, we compile the works where the metabolomics of chlorophylls and/or carotenoids has proved extremely valuable in the recognition or tuning of metabolic pathways correlated with responses to different abiotic and biotic factors; physiologic and biologic studies; and even applications to animal health. In this sense, the aim of the present review is to strengthen the potentiality of the metabolomics studies of chlorophylls and carotenoids. Moving on from an analytical determination, metabolomics is a powerful tool for comprehensive research, with multiple and diverse applications, as will be shown in this review.

## 2. Biochemistry of Chlorophylls and Carotenoids

### 2.1. Chlorophylls

Chlorophylls comprise a homogeneous group of more than 100 different structures with a unique configuration in nature. Their primary function is associated with photosynthesis, being functionals during the charge separation in the reaction centers or transferring energy in the harvesting complex. Unfortunately, this essential role has masked, other actions of chlorophylls in nature, including the interrelation with the general metabolism, and has led to the underestimation of their physiologic functions.

**Figure 2:**
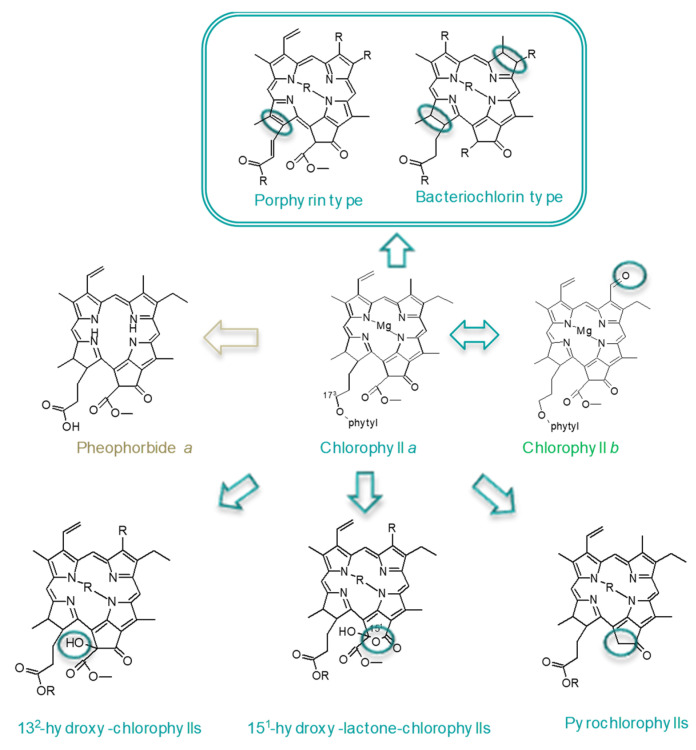
Main chlorophyll structures present in organisms or food due to natural metabolism or during processing or storage.

Chlorophylls are tetrapyrroles with an additional fifth isocyclic ring (Figure 2). They are coordinated generally with a central atom of magnesium, although this can be substituted by hydrogen or other divalent cations. In parallel, the propionic acid at C17^3^ is esterified with a phytyl chain (C_20_H_39_), but different chlorophyll structures arise from esterification with multiple alcohols, and they can even occur in a non-esterified form (as pheophorbides). Chemically, depending on the degree of unsaturation of the macrocycle, chlorophylls could be classified as chlorin type (chlorophyll *a* and *b* among others), porphyrin type (chlorophyll *c*), or bacteriochlorin type (as certain bacteriochlorophylls), which are responsible for a complex array of different chlorophyll metabolites. Moreover, during natural (senescence or ripening) metabolism or food processing or storage, chlorophylls can be oxidized to form new chlorophylls. Among the most common are 13^2^-hydroxy-chlorophylls, which are formed if the hydroxyl group is introduced at C13^2^. In addition, C15^1^-hydroxylactone-chlorophylls are formed if a lactone group is formed at C15^1^, and pyroderivatives are formed if the carboxymethoxy group at C13^2^ is lost.

**Figure 3:**
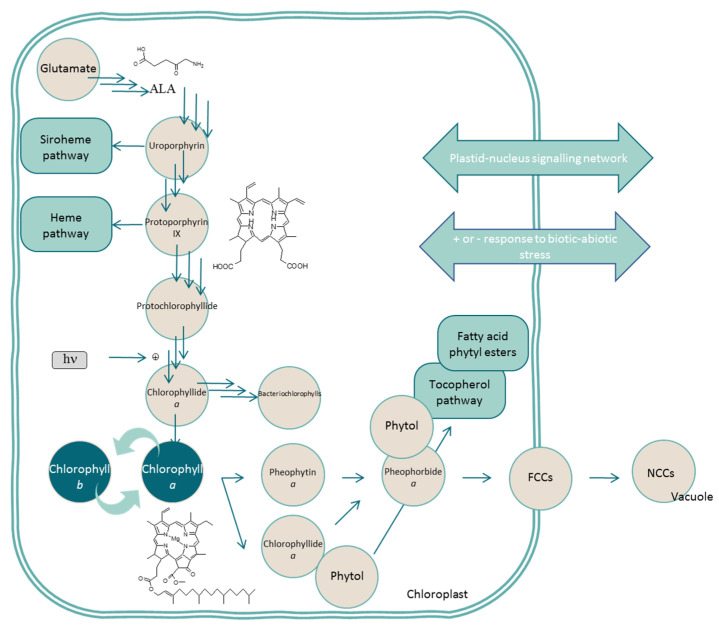
Brief description of the biosynthesis and catabolism of chlorophylls and routes related to other phytochemicals.

Biochemically, the chlorophyll metabolism is divided among synthesis, the chlorophyll cycle, and degradation, and it is independently regulated. Few interesting reviews have detailed the complete set of biochemical reactions, enzymes, and genes implicated in their metabolism [6,7,8,9], and, consequently, we only delineate the main reactions for a general outlook (Figure 3) in this review. Chlorophyll synthesis is initiated from the amino acid metabolism, specifically from aminolevulinic or glutamic acids, depending on the researcher. Different condensation, reductions, and decarboxylations generate protoporphyrin IX, which is the first colored chlorophyll metabolite. This point of the route is a hotspot, as it is where the branch toward heme metabolism occurs if Fe-chelatase inserts Fe in the tetrapyrrole, or, similarly, where the branch toward the chlorophyll metabolism occurs if Mg-chelatase catalyzes the reaction. Following Mg-protoporphyrin IX and after several reactions, protochlorophyllide *a* is formed. This compound is an interesting metabolite because the subsequent reaction is light dependent in angiosperms and thus responsible for the etiolated plants in dark conditions. After several reactions, chlorophylls *a* and *b* are synthesized, with the functional capacity of interconversion through a plastic chlorophyll cycle [9]. Such flexibility in the chlorophyll metabolism is based on the capacity to modify the relative amounts of chlorophyll *a* and chlorophyll *b* depending on the light intensity, modifying the proportion of antenna complexes and, consequently, the photosynthetic apparatus. While chlorophyll synthesis is completely developed in the chloroplast, the catabolic reactions start in the green organelle but run through the cytosol, finishing in the vacuole (Figure 3). Chlorophyll *a* is degraded to pheophorbide *a* in two reactions, liberating the magnesium atom and de-esterifying the phytol chain. Recently, it was demonstrated that phytol yielded from chlorophyll catabolism is essential for tocopherol synthesis [10]. Next, the macrocycle is oxygenolytically opened to form the first linear chlorophyll catabolite, the so-called phyllobilins due to their resemblance to the heme-derived bilins. At present, more than 40 different phyllobilins have been described [11] with unknown functions, although an antioxidant role has been assigned to them. After reduction, a fluorescent chlorophyll catabolite (FCC) is produced and exported from the chloroplast to the cytosol. FCCs could be modified in the cytosol and imported into the vacuole, where the acidic pH promotes isomerization to non-fluorescent chlorophyll catabolites (NCCs). Although a phyllobilin database for *Arabidopsis thaliana* [12] is already available, a complete database containing all phyllobilins identified at present in multiple species is necessary.

As previously stated, in addition to their key role in photosynthesis, chlorophyll compounds are implicated in different physiological actions and biochemical reactions. The photodynamic properties of several chlorophyll metabolites allow them to be implicated in the ROS response [13] and, consequently, as shown below, on different mechanisms, such as defense, stress, and cell death. However, multiple pieces of evidence demonstrate the antioxidant properties of chlorophylls [3]. Another example of the superficial valuation of chlorophylls is the simple determination of chlorophylls as a simple symptom of senescence. If we bear in mind the fact that the physical presence of chlorophylls *a* and *b* is necessary for the assembly of the photosynthetic apparatus, it can be understood that organisms named stay-greens (with a deficiency in senescence) have, in many cases, been identified as mutants in chlorophyll degradation genes.

### 2.2. Carotenoids

Carotenoids are a family of naturally occurring yellow, red, and orange pigments chemically derived from isoprenoids that group together ca. 1200 compounds [14]. Carotenoids are lipophilic compounds synthesized in plastids. In chloroplasts, carotenoids have an essential role in photosynthesis, assisting in harvesting light energy by transferring it to the chlorophylls and protecting the photosynthetic apparatus by quenching triplet excited states of chlorophyll molecules, singlet oxygen, and carboxy radicals [15]. Additionally, they are precursors to phytohormones and other signaling compounds [16,17]. These functions in photosynthesis, photoprotection, and key metabolic pathways make carotenoids essential metabolites. However, the biosynthesis of secondary taxon-specific carotenoids also occurs in chromoplasts, and it is linked with other roles and actions, such as antioxidant activity not being related to photosynthesis and carotenoids serving as intermediates in plant-animal interactions by furnishing flowers and fruits with fragrances and colors [18,19]. Carotenoids with specific structural arrangements are precursors for vitamin A, which has a direct impact on the function of these pigments in human nutrition [20]. Their action as antioxidants and other not yet fully understood activities in mammals have prompted evidence for their role in human health [13]. Furthermore, there is a commercial demand for carotenoids for the food, pharmacy, and cosmetics industries [21]. Altogether, this explains the enormous interest in carotenoid biosynthesis and the possibility of manipulating and engineering the carotenoid biosynthetic pathway to answer fundamental research questions and identify practical applications [22].

**Figure 4:**
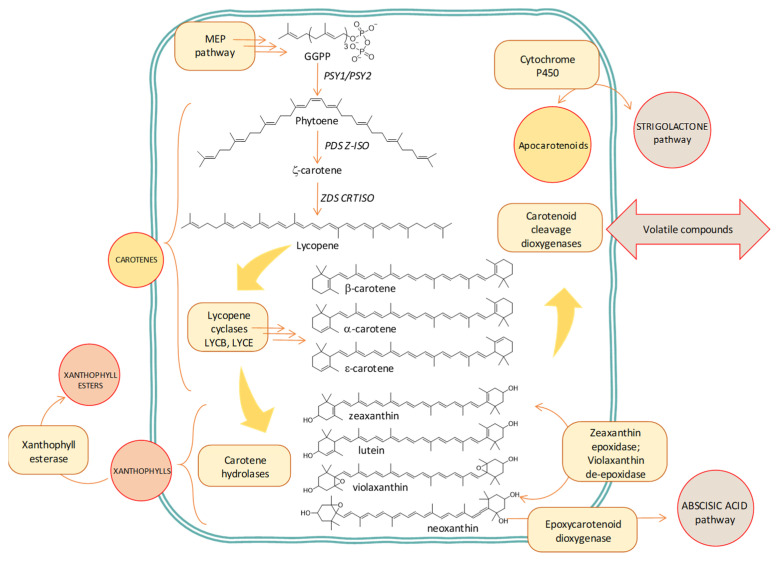
Scheme of the carotenoid biosynthesis route including catabolism to abscisic acid, volatile compounds, and strigolactones. MEP: methylerythritol phosphate; GGPP: geranylgeranyl pyrophosphate; PSY: phytoene synthase; PDS: phytoene desaturase; Z-ISO: ζ-carotene isomerase; ZDS: ζ-carotene desaturase; CRTISO: carotene isomerase.

Carotenoid biosynthesis (Figure 4) starts with a series of isoprene condensations to yield phytoene, a substrate that undergoes desaturation and isomerization steps (yielding a group of intermediates) to form lycopene. These initial steps configure the basic structure that characterizes plant carotenoids: the typical C40 skeleton with a central polyene system that condenses the physicochemical properties of these pigments and conditions and the subsequent enzymatic processes that continue the route [23,24]. From this point, cyclization and subsequent oxygenation of the cyclic intermediates emerge as the breakthrough to the origin of a considerable diversity of carotenoid structures [25]. Different combinations of cyclic arrangements (type β and type ε) at one or both ends of the polyene system generate the first branch in the route, while the introduction of hydroxyl, keto, epoxide, etc. functions produces the classification of carotenoids in carotenes (pure hydrocarbons) and xanthophylls (oxygenated products of carotenes).

At this point, the main issue to consider in metabolome-based studies of carotenoids is the site of carotenoid biosynthesis and accumulation and the structural features that characterize the precursors, products, and catabolites of this family of natural pigments. First, carotenoids are synthesized in plastids and chloro- and chromo-plasts, meaning that compartmentation approaches can be successfully used to focus metabolomics studies and specifically analyze how the pathway operates in this separate location a priori without unexpected competition. Second, the structure of the carotenoids is the premise of solving the analytical challenge of their identification and quantification, while it includes the possibility of expanding the analysis to both parent compounds and metabolic products. Carotenoids present a common basic skeleton (Figure 4), the polyene chain, and a combination of cyclic/linear arrangements at the ends of the skeleton with the introduction of oxygen functions at specific carbon atoms expands the number of structural blends [26]. Moreover, these structural features seem to correspond exclusively to this family of natural pigments. However, the correct identification is only feasible through the acquisition of several layers of information from different technologies (UV-visible spectrophotometry, mass spectrometry, nuclear magnetic resonance, and circular dichroism) combined with a variety of derivatization processes and a comparison with reference standards. The presence of geometric isomers, a frequent feature of carotenoids, complicates the identification task and requires the introduction of secure workflow models and a combination of analytical techniques for successful classification [27,28,29,30].

## 3. The Praxis of Metabolomics: Essential Steps and Challenges for the Experimental Design

**Figure 5:**
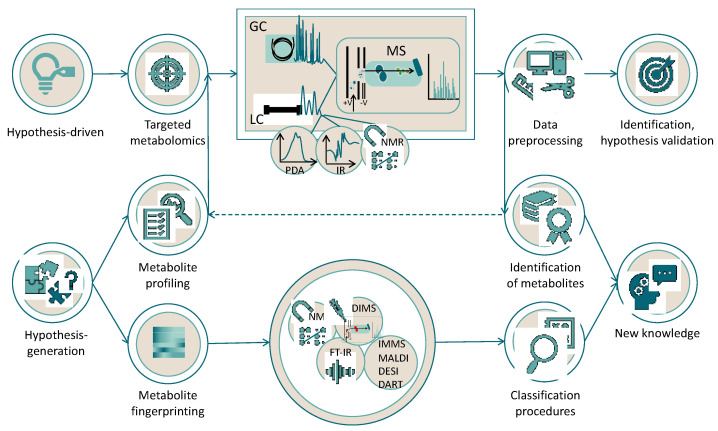
An outlook of the planning of a metabolomics study including the selection of the workflow (hypothesis-driven or hypothesis generation), examples of instrumental techniques, and data preprocessing and interpretation.

When working with chlorophylls and carotenoids, as with other phytochemicals, different metabolomic approaches can be developed (Figure 5). If the goal of a study can be solved with observations and the quantification of a rather limited number of metabolites, which are chosen based on previous literature reports or self-experience, targeted metabolomics is performed. If we encounter a study without a previous hypothesis, then we aim to obtain a global picture of the metabolome, measuring as many metabolites as possible, which means that untargeted metabolomics are suitable. Using this strategy, when samples are classified based on their metabolite profile, without identification of the individual peaks, fingerprinting is carried out. On the contrary, when as many compounds as possible are identified and subsequently quantified, metabolite profiling is carried out.

### 3.1. Material Preparation and Extraction Protocols

In metabolomics studies, frequent potential sources of bias are as follows: an unclear selection of the development stage at harvesting time, a lack of references to provide guidance on the light period and harvest duration, and a lack of a record of environmental variables and growth conditions [31]. This is crucial when working with chlorophylls and carotenoids, because the type of sample handling and applied treatments are critical to avoiding alterations to the metabolites. Additionally, it should be kept in mind that fluxes and accumulation rates are different depending on the class of metabolites (chlorophylls or carotenoids) or the metabolic process (for example, metabolites involved in the photosynthesis, antioxidant activity, catabolism of degradation products during ripening, and tissue senescence) that is focused on [32].

If the analysis of metabolites does not need the pre-processing of the tissue, then the direct flash freezing of the sample in liquid nitrogen stops metabolic conversions, and the frozen sample is homogenized into a fine powder to enhance and standardize metabolite extraction. A significant research effort has been made to refine the protocols for specific chlorophyll and carotenoid extraction, minimizing the sources of errors and increasing the reliability of the data [33]. The experimental design of most protocols aims to reduce the processing time while increasing the efficiency of the extraction. In addition, factors such as economic viability and sustainability have been introduced in the experimental design of those protocols. Therefore, different “green extraction techniques” can be applied, such as supercritical fluid extraction, microwave-assisted extraction, ultrasound-assisted extraction, pulsed electric field extraction, and extraction assisted by enzymes. These techniques have been applied mainly for carotenoid extractions, although several assays were developed for chlorophyll extractions [34]. Supercritical fluid extraction presents several advantages, such as its high purity of the extraction, simplicity, safety, and moderate temperatures [35,36]. On the contrary, it is essential to optimize the temperature and pressure conditions for a specific sample. Better results seem to be obtained when taking advantage of microwave irradiation and when applying microwave-assisted extraction [37,38]. The direct generation of heat within the matrix increases the recovery of the pigments. The studies using ultrasound-assisted extraction showed a significant reduction in the extraction time and an increase in the pigment extraction yields [39]. Pulsed electric field extraction was also used to improve pigments extractions [40,41], but its effectivity depends greatly on the intensity, amplitude, duration, number, and frequency of repetitions. However, besides the excellent results obtained with these protocols, the application of ionic solvents could be considered the most modern extraction methodology at present. An ionic solvent can be defined as compounds completely composed of ions with a melting point below 100 °C. However, additional steps of purification are required when similar structures and/or polarities are present. Therefore, an additional improvement is the set-up of the liquid-liquid extraction process using aqueous solutions of tensioactive ionic liquids and vegetable oil as an alternative to the conventional extraction processes [42], with excellent results for chlorophylls and carotenoids. However, these modern extraction protocols require a considerable amount of time to be generalized, while solvent extraction techniques are the universal protocol applied to obtain chlorophylls and/or carotenoid extracts. The high recovery and stability of the extracted compounds should be poised, and several different solvents are suitable to achieve this aim (methanol, ethanol, acetone, and mixtures at different ratios of water vs. organic solvent(s) at an acidic pH), with the help of sonication and vortex mixing. The selection of the solvent should be made considering the wide range of the polarity of compounds if untargeted metabolomics are pursued, while some solvent mixtures could be tested to extract those compounds of interest selectively for targeted metabolomics. In this case, the appearance of sample matrix effects in the subsequent instrumental analysis diminishes, while interference due to the matrix during analysis and quantification could be a serious issue in the case of metabolite profiling, which requires an almost complete extraction of metabolites.

### 3.2. Technologies: Instrumental Techniques for the Acquisition of Spectroscopic and Spectrometric Data

Once the extract is ready for analysis, the instrumental technique performs the acquisition of a set of data, whose complexity is related to the selected strategy for the metabolomic study (Figure 5). Fingerprinting is typically performed with ^1^H-NMR, ignoring the problem of making individual assignments of peaks [43]. Here, the main issue is to work with signals that are typically evident as multiple peaks, hindering the analysis of data. To overcome this problem, the acquisition of ^13^C-NMR spectra with modern probes and systems purposely created to increase the sensitivity has been noted [44]. With these approaches, the aim is to find a group of marker compounds, which are inferred from shifts of different nuclei that characterize skeletons, aromatic rings, heteroatoms, and typical structural arrangements but are not fully identified. Subsequently, statistical analysis is conducted to classify the samples and draw conclusions based on discrimination, aggrupation, or differentiation of selected variables [45,46]. Technical improvements were made in the last two decades to make definitively the combination of NMR spectroscopy with LC a successful arrangement [47,48] if metabolite profiling or targeted metabolomics is the strategy of the metabolomic study. Metabolite profiling and targeted metabolomics make use of GC, while LC can be applied to targeted and untargeted metabolomics. These techniques of analysis are coupled with one or several detection systems to achieve both the compound separation and detection, collecting spectroscopic and/or mass spectrometric data on individual components of the extract.

GC coupled with mass spectrometry (MS) is a robust chromatographic instrumental technique (Figure 5) with a high compound separation efficiency (peak widths of 2–5 s) that yields reproducible retention times. This feature allows the quick building of spectral libraries of reference analytes that boost the identification of a compound profile in the extract, with a high level of certainty in identification [49]. However, GC is only able for the analysis of thermally stable and volatile compounds (directly from the extract, or once the extract is derivatized to produce volatile products), such as carotenoid degradation products (Figure 4) or phytol (arising from chlorophyll degradation, Figure 3). Additionally, the availability of standards of carotenoid and chlorophyll volatile metabolites is still very limited, so the great advantages of the reproducibility of GC retention times and direct matching with mass spectral libraries are fully usable in metabolomic studies of primary metabolites, which have commercially available standard compounds [50,51,52], but not in pigmentomic studies. Despite these limitations, GC-MS is a suitable technique for the identification of the links between carotenoids, their putative signaling molecules (aroma profile), and the antioxidant potential during fruit ripening, as shown in melon [53], red pepper [54], citrus, and tomato [55], or during the processing of black tea [56] and *Mentha* species [57]. However, the applications remain scarce in the case of chlorophylls [58].

LC emerged from the principles of classic chromatography and the instrumental advances designed for GC, typically used for chlorophylls and carotenoids (Figure 5). The number of possible combinations for mobile phase composition, the increasing amount of packing materials for column building, and the high speed of the cumulative working pressure have definitively improved the efficiency and resolution of this technique, which could be easily combined with a wide range of detection systems in a single workflow [59,60,61]. Liquid chromatography in the classic high-pressure arrangement or the modern ultra-performance technology is typically coupled with different detectors based on optical detection (UV-visible, diode array, fluorescence, evaporative light-scattering, and differential refractive index detectors) applied to carotenoids and chlorophylls in foods [62,63] and biological samples [64,65], or in electrical detection (conductivity, electrochemical, and Corona-charged aerosol detectors), as was shown for the measurement of carotenoid bioavailability [66] and antioxidant potential [67] and in vitamin A equivalence studies [68] in humans. However, while the application of electrochemical detectors for chlorophyll analysis is rather limited [69], the holistic strategy that features metabolomics requires the application of further instrumental techniques to obtain as much information as possible from a single run, so the above-noted detection systems have begun to be combined with infrared, Raman, and NMR spectroscopies. This is the case of the metabolite profiling of microalgae species [70] and vegetable purees [71]. Soft-ionization techniques (electrospray ionization, ESI; atmospheric pressure chemical ionization, APCI) that yield protonated (positive mode) or de-protonated (negative mode) molecular ions are appropriate for the analysis of the most relevant groups of plant secondary metabolites [29,72,73], which are mainly separated with a reversed-phase column providing an efficient retention time and separation index, with a particular emphasis on the detection of isomeric compounds. Usually, APCI is used for carotenoids [74,75,76] and non-polar chlorophylls (chlorophylls and pheophytins) [77,78,79], and ESI is used in the analysis of polar chlorophylls (pheophorbide and chlorophyllide) and phyllobilins [80,81]. However, different configurations of both the ion source and mass analyzer have been implemented, including ion mobility [82] and MALDI [83,84].

To increase the reliability of data in the case of metabolite profiling/targeted metabolomics, where (tentative) identification of pigments is the aim, the acquisition of MS in a high-resolution mode, in combination with tandem MS, is almost a pre-requisite. This combination of working conditions and online experiments allows the analyst to obtain different pieces of information that are conveniently arranged in pairs of independent and orthogonal data of physicochemical properties, facilitating the implementation of workflow protocols for the characterization of chlorophyll and carotenoid metabolic profiles tailored to the selected strategy implemented in the study (targeted metabolomics, fingerprinting, or metabolite profiling) [85].

### 3.3. Application of Different Approaches to Pigment Metabolomics

Table 1 contains some representative work dealing with mass spectrometry in the analysis of chlorophylls and carotenoids that we examine in detail in this section. These studies may serve as the starting point to follow current strategies that successfully enhance the analysis of these plant pigments (Figure 5). The aims of these studies were diverse, so the difficulties and bottle-neck issues that were faced boosted the application of methodological approaches and solutions. Hegeman et al. [86] applied the stable isotope-assisted assignment of elemental composition to constrain the number of potential positive hits for a mass peaking procedure in the identification of chlorophyll derivatives. Similarly, Giavalisco et al. [87] provided a comprehensive multi-isotope labeling-based strategy in combination with a fractionated metabolite extraction protocol to perform unambiguous qualitative and quantitative metabolomics using *A. thaliana* leaf and root extracts.

The characteristic isotopic pattern of the copper chlorophyll derivatives is selected as a fast and specific procedure to characterize precisely the presence of metallo-chlorophyll complexes applied to improve the green coloration of food products [88]. A novel strategy that combines UPLC coupled with traveling wave ion mobility (TWIN) and UV-visible detection is proposed to improve the characterization of chlorophylls and carotenoids analyzed in complex biological matrices [82]. A workflow strategy to perform targeted metabolomics of chlorophyll catabolites is applied to data analysis obtained by HPLC/ESI-hr-QTOF-MS from leaf and fruit senescent tissues [81,89]. Automated data analysis using multivariate curve resolution algorithms to study multi-component systems that follow additive bilinear models (pure spectrum and related time profile) is also an appropriate strategy for the analysis of pigment metabolites. With this method, Wehrens et al. [90,91] examined the metabolite profiles (carotenoids, tocopherols, and chlorophylls) of grapes (*Vitis vinifera*) and cassava (*Manihot escullenta*). Watanabe et al. [92] described a combination of analytic tools that can be used to obtain comprehensive metabolite profiles in the *A. thaliana* plant model. Another interesting approach in the metabolomic studies of chlorophylls is the determination of phytol, a direct metabolite produced by chlorophyll degradation [93] that is analyzed by GC-MS. The incorporation of Bayesian approaches to cluster accessions of *Brassica rapa* of different morphotypes and origins allows for the acquisition of association mapping between different markers and metabolites, including chlorophylls and carotenoids [94]. Authors correct for kinship and population structure with the main aim of reducing the rate of false-positive associations. The implementation of alternative separation procedures, such as supercritical fluid extraction/chromatography coupled with MS, which reduce the extraction time and analysis run time, is an increasingly applied option to achieve a reduction in inter-sample variability and the setting of batch-type applications [95,96].

**Table 1:** Brief description of representative works addressing mass spectrometry in the analysis of chlorophylls and carotenoids.

Raw Material	Extraction Solvent	Instrumental Techniques	Strategy for Metabolomic Study	Ref.
*A. thaliana*	MeOH:H_2_O (8:2)	LC/ESI-TOF	Metabolite profiling based on isotope labeling-assisted elemental composition	[86]
*A. thaliana*	MeOH:MTBE:H_2_O (1:3:1) and subsequent separation with MeOH:H_2_O (1:3)	Multiplatform approach (UPLC-FT-MS and MS/MS, GC-MS, nUPLC-QTOF-MS, and MS/MS)	Metabolite profiling based on isotope labeling-assisted elemental composition	[87]
Olive oil, canned green vegetables	N,N-dimethylformamide	LC/APCI-ESI/hr-QTOF-MS	Metabolite profiling based on isotopic pattern	[88]
Microalgae	EtOH:hexane (2:1) and H_2_O:hexane 1:2	UPLC-UV-TWIM-MS	Untargeted metabolomics	[82]
Lemon (*Citrus lemon* L.)	Acetone	LC/ESI/hr-QTOF-MS	Targeted metabolomics	[89]
*A. thaliana*	Ethanol	UPLC/TOF-MS	Targeted metabolomics	[92]
Wheat (*Triticum aestivum*)	Methanol:acetonitrile:water (4:4:2)	Multiplatform approach (GC-MS, GC-QTOF-MS, LC-MS, and LC-QTOF-MS)	Targeted and untargeted metabolomics	[93]
Tamarillo fruits (*Solanum betaceum*)	CO_2_:MeOH (95:5 or 90:10)	SFE-SFC-MS	Untargeted metabolomics	[96]
Tomato (*Solanum lycopersicum* L.)	MeOH followed by hexane:acetone (1:1)	LC-APCI-QTOF-MS	Metabolite profiling	[97]
*A. thaliana*	Chloroform:MeOH:H_2_O (2:6:2) and derivatization with methoxyamine hydrochloride and N-methyl-N-(trimethylsilyl) trifluoroacetamide	GC-TOF/MS	Metabolite profiling	[98]
*S. lycopersicum* L.	MeOH or MeOH:H_2_O (75:25)	LC-QTOF-MS and LC-PDA-FD	Metabolite profiling	[99]
*Zea mays*	MeOH and dH_2_O with ribitol; derivatization with methoxyamine, N,Obis(trimethylsilyl)trifluoroacetamide, and trimethylchlorosilane	GC-TOF-MS and spectrophotometry	Metabolite profiling	[100]
*S. lycopersicum* L.	MeOH and dH_2_O with ribitol; derivatization with methoxyamine, N,Obis(trimethylsilyl)trifluoroacetamide, and trimethylchlorosilane	GC-TOF-MS and LC-PDA	Metabolite profiling	[101]
*Cucumis melo* L.	Hexane:acetone:ethanol (50:25:25)	LC-PDA	Metabolite profiling	[102]
*Daucus carota*, *Brassica oleracea*, *S. lycopersicum* L.	MeOH:chloroform:Tris-buffer (1.25:1:1.25, 50 mM, pH 7.5)	LC-PDA, LC-PDA-QTOF-MS, GC-MS, and ^1^H-NMR	Targeted and untargeted metabolomics	[71]
*Cuminum cyminum* L.	N,N-dimethylformamide; trichloroacetic acid; chloroform:MeOH:phosphate buffer (1:2:0.9, pH 7.5)	Multiplatform approach (spectrophotometry, LC-PDA, LC-MS, and GC-MS)	Metabolite profiling	[103]
Potato (*Solanum tuberosum*)	MeOH:H_2_O (87.5:12.5)	LC-ESI-QTOF-MS	Metabolite profiling	[104]

## 4. Metabolome-Based Studies of Chlorophylls and Carotenoids

**Figure 6:**
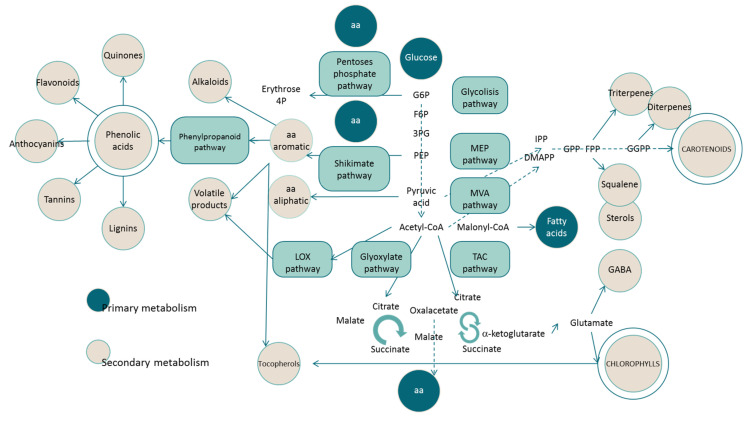
Brief description of some plant pathways and reaction processes that could be related to the biosynthesis and catabolism of chlorophyll and carotenoid pigments.

During the initial development of metabolomics, compounds such as amino acids, organic acids, and carbohydrates were the focus of the studies. However, “pigmentomic analysis” is increasing exponentially, as researchers noticed the metabolic significance of chlorophylls and carotenoids in photosynthetic organisms. Indeed, they are valuable compounds for cells, with physiologic and economic implications. Next, we describe the main applicability areas where the metabolomics of chlorophyll and carotenoids contributes to deciphering a metabolic response. In some cases, the studies integrate metabolite and physiological data with transcriptional information to confirm both molecular and metabolic modifications. Figure 6 presents different pathways that might emerge during a metabolomics study related to chlorophylls and carotenoids.

### 4.1. Application in Abiotic Factors Studies

Biological tolerance is a complex process that includes not only physio-biochemical modifications but also molecular changes. Such metabolic adjustments are required to respond to environmental signals. Consequently, metabolite profiling brings an opportunity to understand the fundamentals of tolerance by searching for modified or different signatures associated with tolerance ability. In this sense, the adaptations of the chlorophyll and carotenoid metabolism of cells exposed to different stresses have been investigated regarding cesium [105], nitric oxide [106], cadmium [107,108], graphene oxide [109], iron [110], nitrogen depletion [111,112,113], and extreme irradiation environments [114,115]. Another research field where the metabolomics of chlorophylls and carotenoids finds successful applications is the study of nanotoxicology, which aims to determine the toxicity of metals and micro- and nano-particles to environmental organisms and how the latter respond to the former. In this regard, it was studied how copper oxide nanoparticles, CuO microparticles, and copper ions perturb the metabolism of aquatics organisms [116] and even the effect of ZnO nanoparticles on the cultivation of terrestrial plants [117]. The most striking advance made in these studies is that the experimental design based in metabolic flux measurements might point out specific responses, which include chlorophyll breakdown and the tuning of carotenoids’ metabolism. These responses reveal metabolomic-based strategies to allow acclimation of the organisms to the factor under study.

As chlorophylls and carotenoids are photosynthetic pigments, there are numerous metabolomics studies regarding the influence of light in the physiology of organisms. In this sense, the metabolomics approach has been applied to investigate the effect of light and dark cycles on the lipid metabolome [118], lineage-specific pathways [119], irradiation-induced stress [120], and photo-regulatory processes [121]. Moreover, metabolomics studies were developed to investigate the influence of LED light on the modulation of the fruit metabolome [122] or the porphyrin and chlorophyll metabolism itself [123]. These studies make use of state-of-the-art comprehensive omics analysis, together with a holistic effective treatment of data, although the translation of the results to the productive field requires further testing.

Another line of research is drought stress, considered to be one of the most important limiting environmental factors for agriculture and responsible for great losses of global food production. Once cells detect water stress, a cascade of signals activates multiple biochemical pathways (Figure 6): hormone induction, gene expression regulation, reactive oxygen species scavenging, carbohydrate and energy metabolism, nitrogen assimilation and amino acid metabolism, fatty acid metabolism, etc. Consequently, high-throughput “omics” techniques are essential to gain a holistic panoramic view of the plant response. In general, transcriptome and metabolite profiling reveals that plants respond to drought by modulating several secondary metabolic pathways and particularly by modifying the production of carotenoids or chlorophylls [124,125,126,127,128], including the extreme example of the adaptation to desiccation, as exhibited by resurrection plants [129].

Regarding salinity, plants have developed several mechanisms to adapt to this stress caused by osmoregulation, such as vacuolar H^+^-ATPases, which are key in cytosol detoxification, as they create an electrochemical H^+^ gradient across the membranes [130]. Transcriptome and metabolome analyses revealed the crucial biological pathways involved in the fast-adaptive response to salt stress, including carotenoid biosynthesis and the metabolism of porphyrin and chlorophyll [131,132,133,134]. An additional multi-omics analysis was used to unveil thermal adaptation strategies of extremophile bacteria [135] and plants [136,137], where the lipid or carotenoid metabolism seems to be implicated. The main effort that requires multi-omics analysis is to select complementary signals in the experimental design, so that the studied signals allow for a deeper understanding of the molecular adaptation of the organism to stress.

A completely different research area where pigment metabolomics was applied is the study of the environmental metabolome, which elucidates the relationship between living organisms and their ecosystem. Through the characterization of the metabolites obtained from the environment, paleometabolites (diagenetic products of chlorophylls and carotenoids derived from photosynthetic algae and bacteria) can be identified [138]. In addition, this technique can be used to determine the toxic effects of organophosphates on the species in freshwater ecosystems [139]. The above compilation is an example of the increasing research areas where chlorophyll and carotenoid metabolomics are involved. This growing trend is broadening our horizons in new, diverse disciplines with a variety of research focuses, such as the determination of metabolic turnover [140], the effects of biostimulants on the metabolome [141,142], and sustainable soil control [143]. All of them are examples of research areas where the metabolomics of chlorophylls and carotenoids has revealed as a successful approach to gather essential information.

### 4.2. Application in Biotic Factor Studies

The interrelation between organisms is a subject that has been scarcely studied, surely due to its complexity. However, it is in this subject where metabolomics could successfully contribute to advances in knowledge because of the inherent capacity of this approach of studying several physiological pathways, responses (Figure 6), and behaviors at the same time. Therefore, through metabolomic studies of chlorophylls and carotenoids, significant advances have been achieved with regard to the interplay between biotic stressors; the effect of single- vs. multiple-pest infestations on the biochemistry of plants [144]; the fluctuation of the leaf metabolome in response to arbuscular mycorrhizal fungi [145]; the microbial networks established during the assemblage of symbiotic microbials, such as in lichens [146]; the ecological interactions that occur at algal surfaces within microbial communities [147]; and the study of the evolutionary origin of symbiosis [148]. In parallel, metabonomic studies have also investigated the interplay between biotic and abiotic stresses, such as the effect of selenium treatments on the oxidative stress response of plants when infected [149] by the enhanced content of chlorophylls and carotenoids and related enzymatic activities. It should be highlighted that this application is very complex, such as the response to interrelation changes at the organ level, while triggering different biosynthetic pathways to down- or up-regulate them.

### 4.3. Application in Physiologic and Molecular Biology Studies

Metabolomics is a powerful tool that can be used not only to analyze the response of photosynthetic organisms to external abiotic or biotic stressors but also to conduct an in-depth investigation of their physiology in the widest meaning of the term. This promising research line with economic consequences is, nevertheless, a complicated area of study, taking into account the multiple variables that are accounted for. Examples of the potentiality of this implementation include the study of the mechanisms controlled by the circadian clock [98] and the effects of the auto-tetra-polyploidy on the balance between the primary and secondary metabolisms [150]. However, the main area of applicability is the behavioral patterns in the accumulation of metabolites (chlorophylls, carotenoids, etc.) paired with specific ripening stages, harvesting periods, cultivars, traceability, and plant tissue functions. Sometimes, such correlations are successfully established despite the genetic background or in a timeline fashion [101,151,152,153,154,155,156]. Additionally, it is possible to distinguish different genetic backgrounds with chemotaxonomic purposes [157], establishing species- and lineage-specific metabolites in marine microalgae [158], and differentiate chemotypes of selected accessions [159]. The metabolomics of chlorophylls and carotenoids could also be used to analyze the effects of postharvest treatments on the metabolism of edible plants [160,161,162,163,164] or for the identification of fast and unequivocal biochemical markers in breeding programs [165,166].

Another field of application is the utilization of metabolomics as a tool to investigate the biochemical pathways implied in the biosynthesis and degradation of these pigments (Figure 3 and Figure 4), identifying pathway cascades [167,168] and revealing the effects of specific genes [169,170,171,172,173]. As a further step, the metabolomics study of pigments could be used as a platform for the development of strategies to engineer fluxes in complex biosynthetic networks [174,175,176]. A subset of pigment-targeted metabolomics is synthetic biology, which combines known molecular components and genes for the implementation of different molecular pathways displaying novel functions and dynamic behavior that do not occur naturally [177]. A workflow that combines gene expression and quantitative metabolomics with mathematical modeling to identify strategies in order to increase production yields of nutritionally significant pigments has even been proposed [178]. This overall approach, although highly informative and practical, could become difficult to apply as a routine method. Lastly, metabolomics is a common and useful approach for identification purposes [57,85,179,180] and the detection of food processing [71,181,182].

### 4.4. Application in Human Health (Health Status, Cancer, Hypertension, and Digestive Efficiency) Studies

In addition to all of these applicability areas, pigment metabolomics has also been applied to the investigation of human health. This is possible thanks to the fact that the concept of health status has moved from just “a state” to “the ability to adapt”, which was denoted as phenotypic flexibility. In this context, metabolomics and proteomics were adopted to correlate micronutrients with the characteristics of metabolic parameters and, ultimately, to health-related processes [183]. A poorly scientifically explored research area is the potential bioactivity of metabolites yielded via the catabolism of chlorophylls and carotenoids. The wide array of catabolic products (Figure 3 and Figure 4), including phyllobilins (bilin-type catabolites of chlorophylls) [184], volatile and non-volatile apocarotenoids arising from the asymmetric cleavage of carotenoids [185], and the carotenoid-derived hormones abscisic acid and strigolactones [186], deserve attention, because they perform antioxidant activities in their natural environment. Additionally, metabolomics was also proposed as a non-invasive and reliable screening technology as an alternative for cancer detection. Currently, diagnostic procedures are costly and invasive, and novel methodologies that could reduce such features of evaluation tests for patients are urgently required [187,188]. Alternative strategies to address the study of cancer are the identification of new compounds against the proliferation of selected cancer cells [189] and the review of the validity of established biomarkers of dietary intake and the identification of novel ones [190]. These studies are still in the hypothesis testing stage and although they embrace a great potential, the focus should be to establish the complex map of cancer-related activities before pointing out a direct link, either positive or negative, between carotenoids and chlorophyll metabolites and cancer effects. 

## 5. Conclusions

Chlorophylls and carotenoids, known antioxidants, are often evaluated in metabolomics studies with regard to the matter under scrutiny (abiotic/biotic stress) and not as a marker of the metabolic status of the organism. Moreover, the evaluation of these plant pigments is performed with instrumental techniques that yield a global profile count rather than via an in-depth description of both the qualitative and quantitative aspects of the pigment catabolites. This review suggests that the assessment of processes for both primary and secondary metabolisms should consider chlorophylls and carotenoids as key contributors to metabolic study and not simply as “signaling” compounds to determine easily whether something is going wrong or well. Thus, the recently increasing number of published papers, summarized in this manuscript, addressing photosynthetic pigments and metabolomics is generating strong expectations for significant advances in our knowledge of metabolomics as a central piece of functional genomics. Indeed, the study of chlorophyll and carotenoid metabolites requires the development of a wide range of protocols, technical applications, and methodologies. This fact reflects the key role of photosynthetic pigments in the plant metabolism, chemotaxonomy, food technology, and animal health.
